# The Role of Biogenic Amines in Social Insects: With a Special Focus on Ants

**DOI:** 10.3390/insects14040386

**Published:** 2023-04-16

**Authors:** Francesca Barbero, Giuseppe Mannino, Luca Pietro Casacci

**Affiliations:** 1 Department of Life Sciences and Systems Biology, University of Turin, Via Accademia Albertina 13, 10123 Turin, Italy; luca.casacci@unito.it; 2 Department of Life Sciences and Systems Biology, University of Turin, Via Gioacchino Quarello 15/A, 10135 Turin, Italy; giuseppe.mannino@unito.it

**Keywords:** dopamine, tyramine, serotine, octopamine, modulation, regulation, social behavior

## Abstract

**Simple Summary:**

Insects, such as wasps, ants, and bees, can live in highly structured societies characterized by a complex organization. The functioning of these societies is achieved through the coordination of several individuals who can be involved in various tasks and whose numbers are regulated to respond to the overall colony status or needs. The regulatory mechanisms of social behavior are not fully unraveled, but molecules such as brain biogenic amines likely play a pivotal role. Here, we review the potential function of biogenic amines such as dopamine, tyramine, serotine, and octopamine in modulating insect social behavior, with a particular emphasis on ants. We discuss the aminergic regulation of the reproductive state, locomotory performance, learning processes, memory, aggression, geomagnetic-driven orientation, and hierarchical or interspecific interactions across groups of Hymenoptera. We also conducted a bibliometric analysis to highlight potential trends and research interest in the literature related to biogenic amines and their potential role as social behavioral modulators. Social insects have proved to be perfect models to further delve into the aminergic regulation of the behavior, and biogenic amines seem to be good candidates as pivotal factors promoting the evolution of sociality in insects.

**Abstract:**

Eusociality represents the higher degree of interaction in insects. This complex social structure is maintained through a multimodal communication system that allows colony members to be flexible in their responses, fulfilling the overall society’s needs. The colony plasticity is supposedly achieved by combining multiple biochemical pathways through the neuromodulation of molecules such as biogenic amines, but the mechanisms through which these regulatory compounds act are far from being fully disentangled. Here, we review the potential function of major bioamines (dopamine, tyramine, serotine, and octopamine) on the behavioral modulation of principal groups of eusocial Hymenoptera, with a special focus on ants. Because functional roles are species- and context-dependent, identifying a direct causal relationship between a biogenic amine variation and behavioral changes is extremely challenging. We also used a quantitative and qualitative synthesis approach to summarize research trends and interests in the literature related to biogenic amines of social insects. Shedding light on the aminergic regulation of behavioral responses will pave the way for an entirely new approach to understanding the evolution of sociality in insects.

## 1. Introduction and Outlines

Sociality is the main trait characterizing insects like bees, wasps, ants, and termites. Thanks to their complex organization, social insects are among the most widespread and ecologically dominant animals worldwide, present in almost all environmental niches. Their success can be ascribed to the strict organization of the colonies, the reproductive strategies, the effective division of labor, and decision-making [1,2]. The most advanced type of sociality and organization is “eusociality”, which is historically grounded on the presence of three main paradigms [1]: (i) Cooperative brood care—the offspring maintenance is shared among various colony members; (ii) Overlapping generations—the coexistence of individuals with different ages or belonging to distinct generations in the same colony; (iii) Reproductive division of labor culminating in the existence of casts. Recently, eusociality has been primarily defined by the reproductive division of labor [3,4].

The social structure is maintained through a complex communication system based on chemical [5,6], acoustical [7,8,9], visual [10,11], and tactile signals [12], as well as benevolent (e.g., trophallaxis or grooming behavior) or aggressive interactions to establish dominance dynamics in the colony hierarchy. Multimodal signals [13,14] are perceived by various sensors and integrated into the central nervous system in the brain of colony members. However, social insects show high flexibility in their responses based on the overall colony status. This plasticity is supposedly achieved by combining multiple biochemical pathways through the neuromodulation of biogenic amines, neuropeptides, and hormones, but the mechanisms through which these regulatory molecules act are far from being fully disentangled [15,16,17].

Among neuroactive compounds, biogenic amines (BAs) are known to function as neurotransmitters in the synaptic gap, neuromodulators of local cell circuits, or neurohormones acting remotely in other body compartments [18,19,20,21,22]. BAs are nitrogen compounds with one (or more) amine groups originating from the decarboxylation of amino acids. An amazing diversity of BAs has been described, with some compounds acting as toxins, while only a few playing a significant physiological role, which differs between vertebrates and invertebrates [20,23,24].

**Figure 1:**
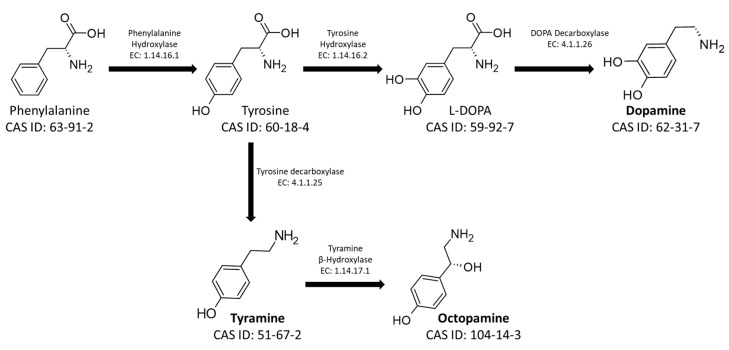
Metabolic pathways for the synthesis of the biogenic amines Tyramine, Octopamine, L-DOPA, and Dopamine. The figure shows the CAS-IDs of the molecules along with the EC numbers of the enzymes involved in the biosynthetic pathway.

The main BAs used as neurotransmitters in social insects are tyramine (TA), dopamine (DA), L-3,4-dihydroxyphenylalanine (L-DOPA), octopamine (OA), and serotonin (5-HT). L-DOPA, DA, TA, and OA are derived from the aromatic amino acid phenylalanine, converted to tyrosine by a hydroxylase (Enzyme Commission number EC: 1.14.16.1). The newly synthesized amino acid can be subsequently converted, according to physiological need, into the four different BAs. Specifically, it is converted into L-DOPA and DA by the action of Tyrosine Hydroxylase (EC: 1.14.16.2) and DOPA Decarboxylase (EC: 4.1.1.26). Otherwise, it is transformed into TA and OA by Tyrosine Decarboxylase (EC: 4.1.1.25) and Tyramine Hydroxylase (EC: 1.14.17.1), respectively (Figure 1) [25,26].

**Figure 2:**
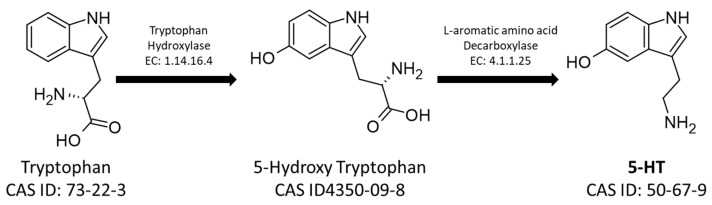
The metabolic pathway for the synthesis of serotonin. The figure shows the CAS-IDs of the molecules along with the EC numbers of the enzymes involved in the biosynthetic pathway.

On the other hand, the 5-HT pathway starts from the aromatic amino acid tryptophan, which is hydroxylated by Tryptophan Hydroxylase (EC: 1.14.16.4) into 5-hydroxytryptophan, which is consequently decarboxylated by L-amino-acid decarboxylase producing 5-HT (Figure 2) [27,28].

In the last decades, the aminergic regulation of social behavior in insects has been increasingly investigated. Here, we review the potential function of major BAs on social behavior in principal groups of eusocial Hymenoptera belonging to ants, bees, and wasps. We cursorily discuss the aminergic regulation of the reproductive state while focusing on the substantial effects of BAs on locomotory performance, learning processes, memory, aggression, geomagnetic-driven orientation, and hierarchical or interspecific interactions across taxonomic groups, with a particular emphasis on ants [29,30,31,32,33,34,35,36]. These functions are species- and context-dependent; thus, identifying a unique role for each BA is extremely challenging. In addition, because most of these findings are the outcome of correlative studies and the direct causal effect of BAs on modulating insect behavioral responses or the sensory pathways affected by these molecular regulators are still to be wholly unraveled, we discuss future perspectives and research roadmaps [17].

Our review aims to prompt researchers to delve into the proximate and ultimate mechanism of BA regulation on social behavior. Studies that shed light on the association between BAs and behavioral responses will pave the way for an entirely new approach to understanding the evolution of sociality in insects.

## 2. Bibliometric Analysis

In this section, a quantitative and qualitative synthesis approach was used to review published evidence in the literature related to BAs and their potential role in social insects. A traditional bibliometric study is an essential step before conducting any evaluation using the most recent bibliometric and meta-analysis approaches [37]. In particular, bibliometric analysis allows for summarizing research trends and identifying research interests, also using author affiliations as potential variables. In addition, it can be used to provide a comparative analysis of scientific productivity at the country, institution, or individual level [38].

Consequently, to deal with these purposes, an extensive exploration was conducted using “biogenic amines AND social insect” as a search string in the Web of Science, PubMed, Scopus, and Google Scholar databases, allowing the identification of 964 entries. Only articles already published in international journals after rigorous anonymous refereeing were included to optimize the construction of the database. In addition, to restrict the search to the most recent scientific findings, manuscripts published before 2014 were excluded (time interval 2014–2023). The required language was English, and the selected manuscripts had to be accessible for online inspection. Finally, punctuation, marks, and singular/plural forms were ignored. As a result, 201 entries were identified and included in the bibliometric analysis.

**Figure 3:**
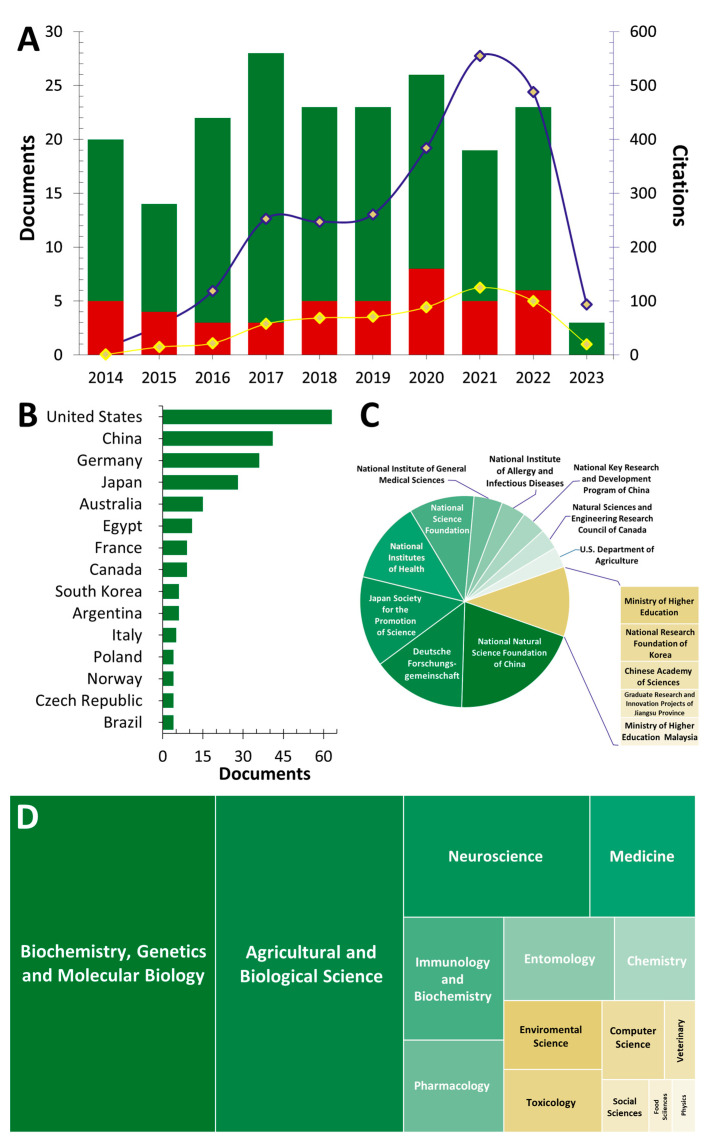
Representation of bibliometric data derived from 201 articles included in the database and obtained using “Biogenic Amines AND Social Insects” as the search string. Panel (**A**) shows the trend of publications and citations in the time interval 2014–2023 (green bars and blue line). In the same panel, the red bars and the yellow line refer to publications related to ants. Panel (**B**) lists the leading countries of authors by the number of publications. Panel (**C**) shows the affiliations of the authors who contributed the most to the research topic. Panel (**D**) shows the scientific fields involved in the topic of interest.

The information contained in the title, abstract, and keywords of each selected article was downloaded in comma-separated values (.csv) format and used for bibliometric evaluations (Figure 3).

Interest in biogenic amines and their potential role in social insects was first dated in 1967 through a publication by Rothbailer A.B. entitled “Aggression, defense and neurohumors” [39]. However, due to the lack of knowledge and the necessary tools to investigate and confirm the involvement of BAs in social insects, the topic never created a genuine interest in the scientific community, as evidenced by the low number of articles published in the years after 1967 and before 2014 (*n* = 20 between 1967 and 2004, and *n* = 35 between 2004 and 2014) but also by the low citation number recorded in 2014 (Figure 3A, trend line).

Regarding the time range covered in this section, the number of articles related to the research topic did not significantly vary between 2014 and 2022, registering a median value of 20.10 ± 7.21 articles per year (Figure 3A, green bars). The highest peak, with 28 studies, was observed in 2017, while 23 articles were published in 2018 and 2022 (Figure 3A, bars). However, a different trend was observed for the citation count (Figure 3A, blue line). Indeed, from the 14 total citations recorded in 2014, an almost exponential increase was observed until 2021, when 555 citations were recorded (Figure 3A, blue trend line). A slight decrease in citation numbers occurred in 2022 (*n* = 488), but this year still exceeded the citations recorded in 2021 or previous years (Figure 3A, blue trend line). On the other hand, analyzing only ant-related publications (Figure 3A, red bars), an average of about four articles per year was recorded, with a citation trend mainly increasing (Figure 3A, yellow trend line). This finding suggests that ants are less investigated than bees as experimental models to study the role of BA in behavioral changes. However, because of the increase in citations, it is arguable that the scientific community’s interest in this topic is constantly increasing.

Figure 3B shows the countries with the best publication results related to BA and social insects, while Figure 3C shows the main affiliation of the authors contributing to the research topic. The analysis revealed that the dominant country in the global scenario having the highest number of publications is the United States, with more than 25% of the total articles during the examined time range. However, China and Germany have also published an increasing number of papers in the field over the past decade, each recording about 15% of total publications. These countries are followed by Japan, which has published 28 articles in 10 years. All other countries registered publication rates of about 5% or significantly lower (Figure 3B). At the European level, besides Germany, it would suggest that exclusively France, Italy, and Poland are conducting research activities related to the topic of interest, reporting 3.67%, 2.04%, and 1.63% of the total publications, respectively (Figure 3B).

Biochemistry, Genetics, and Molecular Biology are the most frequently involved research areas (Figure 3D), accounting for more than 26% of the total publications. Agricultural and Biological Sciences follow closely, resulting as the second disciplinary sectors, with a slightly lower proportion of the publication total (24%). In addition, Chemistry, Entomology, and Neuroscience each collect about 8%. This wide distribution of scientific research areas, together with the reasonable percentage recorded for entomology, suggests not only the presence of solid collaboration among authors with different backgrounds but also the use of multidisciplinary approaches to investigate the research topic, including biochemical and biomolecular methodologies.

## 3. VosViewer Analysis

The database used for bibliometric analysis was further processed to perform a graphical analysis through VosViewer software [40]. This software is a computational tool that provides a fully automated analysis of the terms in the examined manuscripts’ titles, abstracts, keywords, and affiliation sections. The text-mining function makes it possible to visualize networks between different terms, establish semantic clusters, and the terms’ occurrence also from a temporal perspective [41].

**Figure 4:**
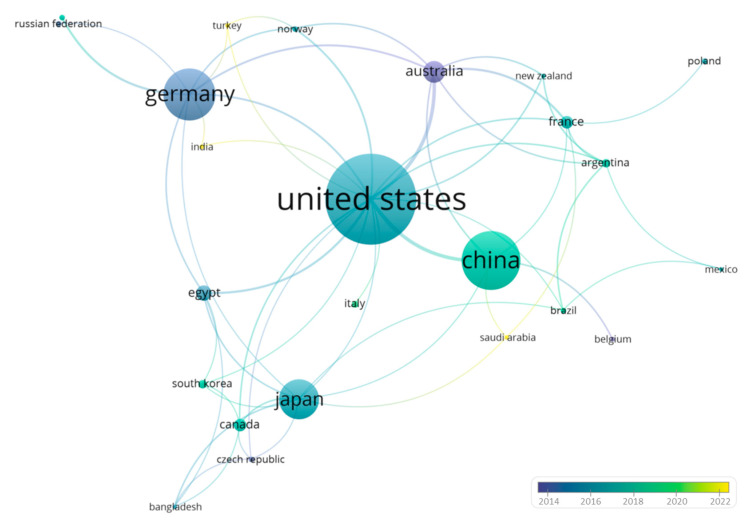
Network Mapping showing the existing co-authorship relationships among countries involved in the publication of scientific articles related to the role of biogenic amines in social insects. The blue-dark bubble is associated with the country when the involvement is less recent, while the yellow bubble refers to countries that have contributed more recently to the research topic. Finally, the size of the nodes indicates their frequency, while the curves between the nodes represent their co-authorship in the same publication.

Here, to further investigate the data shown in Figure 1B, we first carried out a co-authorship analysis to comprehend the potential interactions between the different countries, investigating the role of biogenic amines in social insects over the past decade. The full-counting method was used for this analysis, and documents with a larger number of authors were ignored (*n* > 25). Moreover, the minimum number of documents and citations of an author was set at five and two, respectively. For each of the selected authors (*n* = 34), the total strength of the co-authorship links with other authors was calculated (Figure 4).

Figure 4 confirms the data reported in Figure 3B, showing the United States (Link: 10; Total Link Strength: 36; Documents: 63; Average Publication Years: 2016) as the leading country in publishing scientific articles related to the research topic. The overall top ranking was also confirmed, reporting China (Link: 4; Total Link Strength: 14; Documents: 41; Average Publication Years: 2018) and Germany (Link: 4; Total Link Strength: 9; Documents: 36; Average Publication Years: 2015) at almost equal footing, followed by Japan (Link: 6; Total Link Strength: 8; Documents: 28; Average Publication Years: 2017) and Australia (Link: 5; Total Link Strength: 21; Documents: 15; Average Publication Years: 2015). Interestingly, although India, Turkey, Saudi Arabia, and Italy statistically published fewer papers than the United States and Germany, they have a significantly higher Average Publication Year Score. Specifically, India, Turkey, and Saudi Arabia claim a score of 2022, while Italy equals 2019. This finding strongly suggests that interest in the research topic has drastically decreased in America and Germany while exponentially increasing in the latter geographic locations.

**Figure 5:**
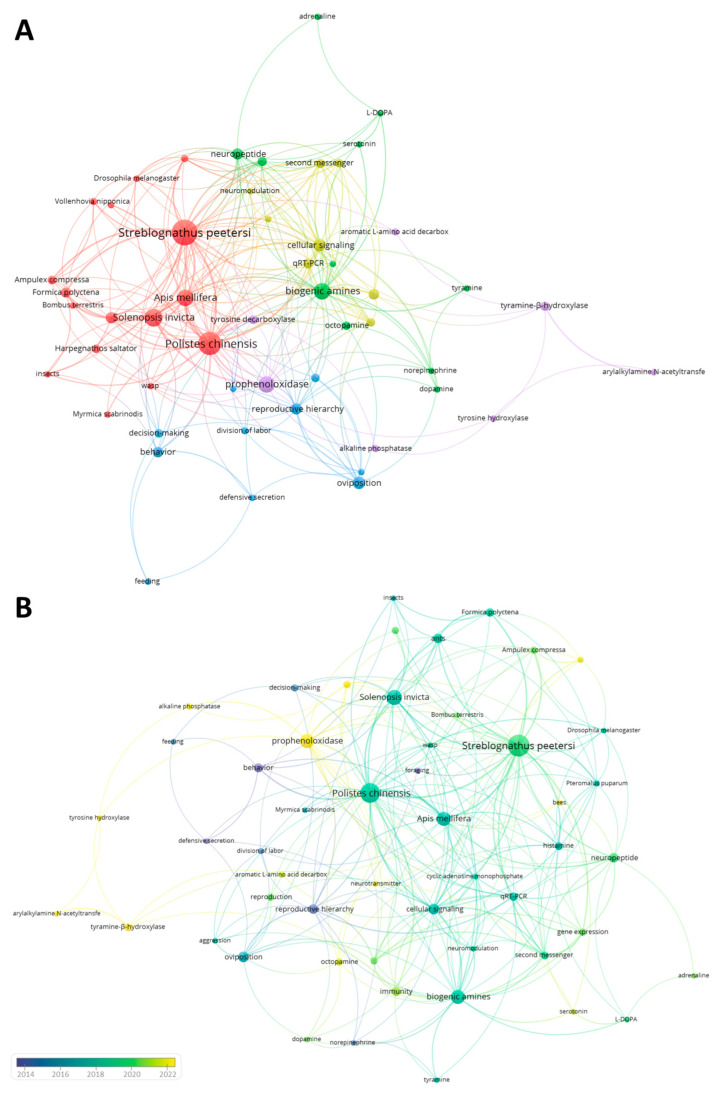
Co-occurrence map showing the existing networking of terms reported as keywords in publications related to the role of biogenic amines in social insects. Panel A shows the grouping of the selected terms into five different semantic groups (cluster I: red dots; cluster II: green dots; cluster III: blue dots; cluster IV: violet dots; cluster V: yellow dots). Panel B shows the same network from a temporal perspective. The blue-dark bubble is associated with terms when their involvement is less recent, while the yellow bubble refers to words that have been used in recent times. For both panels, the size of the nodes indicates their frequency. The curves between the nodes represent their co-occurrence in the same publication.

A co-occurrence network mapping was also constructed using the author and index keywords present at least five times in both title, abstract, and keywords section. In addition, plural/singular forms, punctuation marks, and/or synonyms were adjusted and manually unified. In this case, a whole counting method was employed, and of the identified 2500 terms, the 52 most frequently recurring ones were plotted and clustered (Figure 5).

Cluster I (Figure 5A, red dots) contained 16 items sharing the family and species of insects used for experiments. The most recurrent mentions are related to wasp species (total link strength: 129; total links generated 68; occurrence: 129), and in particular, *Polistes chinensis* (total link strength: 80; total links generated 6; occurrence: 34). Among the used terms also recurs bees such as *Apis mellifera* (total link strength: 45; total links generated 19; occurrence: 22) and *Bombus terrestris* (total link strength: 13; total links generated 13; occurrence: 13). Finally, a remarkable contribution was also given from ants, and the most frequently mentioned are *Streblognathus peetersi* (total link strength: 34; total links generated 24; occurrence: 40) and *Solenopsis invicta* (total link strength: 40; total links generated 19; occurrence: 22).

Cluster II (Figure 5A, green dots) comprised 11 terms, mainly related to molecules with potential neuromodulator activity. The most frequently recurring terms were ‘biogenic amines’ (total link strength: 129; total links generated 68; occurrence: 129) and ‘neuropeptide’ (total link strength: 88; total links generated 48; occurrence: 99). As far as hypothesized from the current scientific literature, the behavior of social insects is mainly influenced not only by molecules derived from decarboxylation of amino acids (biogenic amines) but also by small molecules composed principally of short amino acid sequences named neuropeptides [22]. However, the current knowledge related to neuropeptides is very limited compared to biogenic amines, and several of these molecules are still unknown, and their chemical structure is only hypothesized [32]. The other terms included in this cluster are biogenic amines, and their occurrence suggests the major interest in studying a specific compound. For example, octopamine (total link strength: 10; total links generated 5; occurrence: 10), dopamine (total link strength: 10; total links generated 5; occurrence: 10), tyramine (total link strength: 8; total links generated 4; occurrence: 6), and serotonin (total link strength: 9; total links generated 4; occurrence: 8) are the molecules that showed the highest frequency and are involved in most interactions with terms from the other clusters.

Cluster III (Figure 5, blue dots) contained terms related to social insect behaviors that were evaluated in correlation with biogenic amines. These terms included recurring terms such as ‘reproductive hierarchy’ (total link strength: 9; total links generated 14; occurrence: 25), decision-making (total link strength: 6; total links generated 6; occurrence: 8), reproduction (total link strength: 5; total links generated 5; occurrence: 5), division of labor (total link strength: 3; total links generated 6; occurrence: 6), aggression (total link strength: 7; total links generated 3; occurrence: 6), foraging (total link strength: 4; total links generated 4; occurrence: 4), and feeding (total link strength: 3; total links generated 3; occurrence: 3). Some of these functions will be described and discussed with more detail in the next section (see Section 4).

Clusters IV and V (Figure 5, yellow and violet dots, respectively) contained terms related to biochemistry and molecular biology. In particular, Cluster IV included targets that were probably investigated to study potential mechanisms of actions involved in the research topic. Consequently, entries such as ‘g-protein coupled receptors’ (total link strength: 4; total links generated 20; occurrence: 12), cellular signaling (total link strength: 22; total links generated 43; occurrence: 15), cyclic adenosine monophosphate (total link strength: 7; total links generated 7; occurrence: 3), gene expression (total link strength: 10; total links generated 26; occurrence: 6), and second messenger (total link strength: 4; total links generated 13; occurrence: 28) were reported. On the other hand, in cluster V were mainly found enzymes involved in the biosynthesis of biogenic amines, including alkaline phosphatase (total link strength: 4; total links generated 4; occurrence: 4), tyramine-β-hydroxylase (total link strength: 5; total links generated 5; occurrence: 7), tyrosine decarboxylase (total link strength: 14; total links generated 13; occurrence: 28), arylalkylamine N-acetyltransferase, (total link strength: 5; total links generated 11; occurrence: 12), aromatic L-amino acid decarboxylase (total link strength: 5; total links generated 2; occurrence: 4), prophenoloxidase (total link strength: 17; total links generated 32; occurrence: 14), and tyrosine hydroxylase (total link strength: 4; total links generated 4; occurrence: 5).

## 4. Functional Role of Biogenic Amines in Social Insects

### 4.1. Reproduction and Castes

An increasing number of works suggest that the switch from the non-reproductive to reproductive phase in social insects is correlated with an upturn titer of dopamine in the individual brain. This variation has been observed in several species, such as honeybees [42], bumblebees [43], and *Polistes* wasps [44,45]. The potential outcomes of such an increase in dopamine have been delved deeper into honeybees and related to other functions in which this BA is recognized to be involved. For instance, it has been suggested that the higher dopamine content measured in the virgin queens could increase their aggressiveness, thus endowing them with an advantage during fights with the other queens [46,47]. Because dopamine is also known to promote muscle activity and, ultimately, locomotor behavior (see Section 4.2 and 4.3), high levels of this BA in virgin honeybee queens have also been correlated to the promotion or enhancement of mating flight [48,49].

Peculiar study models are provided by queenless species of ants, in which morphological distinction between the reproductive and the non-reproductive caste is lacking, and a dominance hierarchy maintains the social organization. Through ritualized fights, the dominant females become reproductive while the vast majority of workers remain infertile. Reproductive dominance and hierarchical plasticity proved to be strictly linked to brain neurochemistry, as shown in the ponerine ants *Streblognathus peetersi* [50]. Unique behavioral patterns allow us to identify three groups of ants in this species: (i) the dominant, called alpha, is the one mating and laying eggs; (ii) the high-rank workers, submitted to the alpha but dominant on (iii) the low-rank workers. The alpha workers were characterized by significantly higher levels of octopamine, while serotonin and dopamine did not consistently vary with the reproductive activity in *S. peetersi* [50]. As it occurred in honeybees, it is not clear if these BAs variations are strictly linked to the reproductive maturation process or are more devoted to enhancing behavioral changes, such as an increase in aggressiveness, which is needed to foster dominance hierarchies. Both scenarios are likely and not mutually exclusive. Some direct evidence of the role of BAs in ants’ physiological shift to reproduction has been reported by Penick and colleagues [51], that observed an increase in dopamine levels correlated with ovarian activity in *Herpegnathos saltator*. Indeed, a rise in dopamine content was highlighted when workers with a functional spermatheca, namely gamergates, start reproducing sexually [51,52]. In *H. saltator*, three types of dopamine receptors were located on the ovary tissues of the gamergates, although the ovarian expression levels of dopaminergic receptor genes were lower than those observed in the brain. Fertility and dominance decreased as the dopamine levels dropped as a consequence of worker policing, a social mechanism used to diminish ovarian activity and to control the number of gamergates. Consequently, a decrease in dopamine may be the first event leading to reduced ovarian activity. Therefore, it is also likely that the queen pheromones used to prevent the development of other queens target similar receptors, eventually decreasing dopamine contents. In *Solenopsis invicta*, the pheromone released by the queen inhibits the dealation and reproduction of virgin females by acting on dopamine levels. Indeed, the levels of this amine in the brain of virgin females doubled after only 15 days of separation from the queen of the natal colony [53]. In addition to *Solenopsis* ants [53,54], the honeybee queen pheromone is also known to modulate worker responses by regulating the contents of their BAs, primarily dopamine [55,56].

Unexpectedly, significant differences in the BA levels between queen, male, and worker castes are inconsistent throughout all social insects. For instance, while studying *Formica japonica*, Aonuma and Watanabe [57] did not find any variation in the average contents of dopamine among castes, while higher titers of this BA were detected in *Bombus* [58] and honeybee [59] queens than in workers. Interestingly, changes in brain morphology, which can be correlated with variations in BA contents, were shown in males of *H. saltator* ants [60]. Males possessed a smaller brain than workers, but optic lobes and central complex neuropils were over-developed. In addition, these two regions contained high densities of serotoninergic processes while the rest of the male brain is innervated by fewer aminergic fibers than that of workers. Therefore, in males, serotonin was primarily concentrated in these two brain regions. The peculiar neuroanatomy could provide males with an enhanced ability to perceive visual cues and coordinate movements which are crucial for a successful nuptial flight [60].

Although further corroboration is needed, it is likely a combination of caste interactions, releasing of chemical compounds such as pheromones, but also specific diet (see the royal jelly encompassing precursors of dopamine) [47,61] could act as potential modifiers of BA brain levels (primarily dopamine) which ultimately drive reproductive changes in social insects [54,55,56,57,58,59,62].

### 4.2. Foraging, Aging, and Labor Division

The hallmark of eusocial species is the strict division of labor among colony members. Temporal polyethism is a common mechanism of functional specialization that consists of splitting tasks based on worker age. In general, the youngest individuals are invested primarily with indoor duties (such as brood care) while, as they get older, they face a gradual transition to extranidial activities. Outdoor tasks are connected with higher risks but also require processing complex or multiple stimuli using more advanced computational, locomotory, and biomechanical capabilities [63,64] and references therein. Variations in BA levels were identified as one of the leading causes of behavioral plasticity and social insect specialization in different tasks. As it occurred in the case of maturation between non-reproductive and reproductive states, most of the studies reported an increase in the content of certain BAs as aging. An upturn in three amines, i.e., dopamine, octopamine, and serotonin, was found in bees [65] and ants [51,52,66] as they grew and developed. However, when Wnuk and collaborators [67] surveyed differences in BAs according to age, task specialization, and behavioral maturation in *Formica polyctena*, they found that the octopamine levels were significantly higher in nurses, which are younger individuals, than in foragers, being partially in contrast with the aforementioned findings. Overall, *F. polyctena* proved an interesting model because the division of labor associated with sub-castes in this species can be modified according to the colony requirements. Thus, it is likely to observe a behavioral change in foragers that return to in-nest duties, the so-called “reverted nurses”. Since the amine contents did not differ significantly between “reverted nurses” and foragers, the authors concluded that BAs in this ant species are maturation-related rather than task-related [67]. Nonetheless, a study on *Acromyrmex echinatior,* a leaf-cutting ant species, established a strong correlation between the division of labor and monoamine brain variations [68]. *A. echinatior* shows a very complex task specialization with a remarkable morphological distinction between workers. Foragers are bigger ants who leave the nest searching for leaf material to grow their symbiotic fungi, whereas waste-managing workers are smaller individuals that remove leftovers and infectious material from the nest. Foragers showed higher levels of octopamine and dopamine, while serotonin contents were similar to those of waste-managing workers. These results align with studies on other social insects, where octopamine was the main neurotransmitter involved in olfactory memory and outdoor activities [69,70]. High concentrations of octopamine may be related to the foraging activities because they require encoding several olfactory stimuli, like pheromone trails left by nestmates or identifying the food source and quality. Indeed, the involvement of octopamine, and partially of dopamine, in sensing pheromones in the contest of reward learning was also found in Hymenoptera, primary honeybees [71,72,73,74]. Recently, Baracchi and colleagues [74] found that the effect of attractive compounds, such as geraniol, on learning and memory was modulated through octopaminergic and dopaminergic signaling. The most robust evidence of BA involvement in chemical sensing in ants was provided by studying *Pheidole dentata*. In this species, serotine mediates the forager individual’s response to pheromone trails [75]. 

Pheromones are not the only signals social insects use while foraging; other cues can be pivotal to finding food resources and returning to the nest. By investigating the foraging behavior of *Lasius niger* ants, Mannino and co-workers [29] highlighted that the geomagnetic field plays a crucial role in enhancing the workers’ orientation performance. With respect to workers assayed in near-null magnetic field conditions, ants kept in the presence of a normal geomagnetic field showed significantly higher contents of several BAs, which could explain the increase in locomotory (tyramine and dopamine) and chemical perception (octopamine and serotonin) abilities observed during behavioral tests. This work proves that BA modulation could be the molecular mechanism through which the geomagnetic field affects *L. niger* orientation performance.

### 4.3. Nestmate Recognition and Aggressive Behavior

In social insects, discriminating between nestmates and non-nestmates is a fundamental process for defending their societies and territories from members of other colonies or species. Within a colony, each individual possesses a layer of several compounds, especially cuticular hydrocarbons, which primarily protect the insect from desiccation, but have become essential communication signals during evolution [6]. Trophallaxis and allogrooming permit the transfer of these compounds among members of the society, allowing to assemble the so-called “colony odor” [76].

Two main hypotheses have been formulated to explain the nestmate discrimination mechanism in ants. The first foresees that each colony member can perceive its own chemical signature, and during the encounter with another individual, it compares with the chemical signature of the encountered subject [77]. The second hypothesis is that ants acquire the odor of their colony and nestmates in a continuous learning process to meet any changes in the chemical profile over time [78]. Regardless of the recognition mechanism, when an individual recognizes another individual as a stranger, they will engage in a series of defensive behaviors [79]. 

Several studies have highlighted the active involvement of biogenic amines in the intra- and interspecific recognition process and in aggressive reactions. In honeybees, for example, octopamine-treated workers increased aggression toward non-nestmates and reduced aggression toward nestmates, indicating that higher octopamine concentrations improve recognition of nestmates by reducing errors and increasing attention to relevant sensory stimuli [80]. Furthermore, when stimulated with isoamyl acetate, the main component of the alarm pheromone, the levels of two other biogenic amines, serotonin and dopamine, increased, inducing a greater defensive reaction, e.g., stinging behavior [81].

In ants, several biogenic amines appear to be involved in intra- and interspecific recognition processes and aggressive reactions. In *Tetramorium caespitum*, for instance, changes in octopamine and serotonin in individuals’ brains are sufficient to modify worker ants’ decision to attack non-nestmate individuals, while increases in dopamine levels are associated with physical combat [82]. In workers of *Formica aquilonia* fed with octopamine, the frequency of attacks toward their natural predators, i.e., ground beetles, increased while the frequency of non-aggressive reactions decreased [83]. In a congeneric species, i.e., *F. polyctena*, biogenic amine administration was demonstrated to have significant context-dependent behavioral effects, being almost exclusively exhibited in the case of confrontations with allospecific opponents (*F. fusca* and crickets’ nymphs). Serotonin treatment produced a weak stimulatory effect on aggressive behavior patterns, while dopamine elicited several strong aggressive behaviors toward the opponents. On the contrary, octopamine did not cause any significant effects on the aggressive behaviors of the tested ants, while tyramine administration suppressed threatening behavior directed at *F. fusca* [84].

The work by Aonuma [85] on *Odontomacus kuroiwae* shows how levels of biogenic amines can vary at the individual level influencing behavioral patterns related to aggression and defensive responses. Among workers of the trap-jaw ant, less than 10% of individuals tactilely stimulated at the level of the abdomen responded with a rapid turn indicating a higher efficiency in defense behavior than most workers that escape after the stimulus. The levels of octopamine, dopamine, and serotonin in the brain of the workers of the first group were significantly elevated, and the supplementation of serotonin and moderate dopamine contributed to the triggering of defensive responses. A difference in aggressive behavior at the individual level has also been found in *Oecophylla smaragdina* [86], in which polyphenism exists as in many other ant species; the major workers defend the territory while the minors take care of the offspring. Octopamine modulates aggression in the two sub-castes; levels were significantly higher in major workers, and octopamine was positively correlated with the frequency of aggressive responses to non-nursery mates, indicating that neuromodulators may be associated with size differences and associated specializations.

### 4.4. Trophallaxis, Feeding, and Interspecific Interactions

Trophallaxis is the exchange of food, nutrients, and other fluids between colony members, i.e., between workers, between workers and queens, and between workers and larvae. It occurs with the exchange of liquids through mouth-to-mouth contact (stomodeal trophallaxis) or anus-mouth (proctodeal trophallaxis). Trophallaxis has an essential role in the establishment of social bonds, in maintaining a balance in the food flow between colony members, and in recruiting new young members of other conspecific colonies [87,88].

In ants, trophallaxis and grooming are typical social behaviors shared among nestmates. In a study by Wada-Katsumata and colleagues [89] on *Formica japonica*, seven-day food deprivation reduced dopamine levels in workers’ brains, and the starved individuals did not perform trophallaxis toward nestmates. The brain octopamine level tended instead to increase in those individuals who were isolated from nestmates. Normal levels of the two biogenic amines were re-established when the isolated ants were reintroduced into the colony, indicating that social interactions, e.g., trophallaxis and grooming, with nestmates can influence the homeostasis of brain biogenic amines in *F. japonica*. Similarly, in the ant *Camponotus fellah*, octopamine reduced trophallaxis in workers, indicating that this molecule could be partly responsible for social cohesion among nestmates in ant colonies [87]. In starved individuals of *Formica japonica*, Wada-Katsumata and colleagues [89] observed a reduction in the frequency of trophallaxis and dopamine levels. After sucrose feeding, dopamine levels remained low or even decreased. In contrast, hungry subjects restored normal dopamine levels after the trophallaxis occurred, confirming that this particular interaction not only improves social bonding but also helps to modulate stress in individuals, thus maintaining the colony balance. 

Another biogenic amine may be involved in the feeding process. Indeed, it seems that serotonin can induce satiety in the *Componotus mus* ant by lowering the intake of carbohydrates. Individuals given oral serotonin and sucrose showed reduced interest in carbohydrates while preferring to feed on protein solutions [90]. However, it is impossible to say whether this change is caused directly by serotonin itself or by the sucrose solution; thus, further investigations are needed [90].

BAs were shown to play a role also in interspecific interactions. Recently, some associations previously defined as mutualistic have been reviewed as potential cases of host manipulation. An outstanding example is provided by the work of Hojo and colleagues [91], who found that the secretions released by a butterfly caterpillar, *Narathura japonica*, increased the aggressiveness and reduced the locomotory ability of the host ant *Pristomyrmex punctatus* by regulating the dopamine levels in the worker’s brain. Therefore, the ants offer protection to the butterfly larvae not because of the nutritional gain provided by their secretions but because through these, they receive manipulative drugs acting on their behavior, eventually enhancing their partner fidelity and guarding. 

A similar scenario was described in an aphid-ant system, but here the manipulation occurred in both directions. *Lasius japonicus* ants received high-quality food rewards by promoting the reproductions of particularly honeydew productive morphs of *Macrosiphoniella yomogicola* [92]. On the other hand, the aphids controlled host ants by secreting dopamine droplets, increasing their aggressiveness. Therefore, *M. yomogicola* manipulates ants by means of dopamine to gain better protection [92].

A third example involved the interaction between plants and ants. Studying *Myrmica scabrinodis,* the authors found that a blend of *Origanum vulgare* terpenoids, i.e., carvacrol and thymol, decreased the locomotor activity of ants and increased their aggressiveness by regulating the BA levels in workers’ brains [24]. The release of carvacrol is triggered by the interaction between the plant and *Myrmica* ants, and this chemical signaling is eavesdropped by females of a myrmecophilous butterfly, *Maculinea arion*, to locate their optimal egg-laying site [93]. *M. arion* is an obligate social parasite of *Myrmica* ants and scent carvacrol to spot *Origanum* foodplants growing in the foraging range of a *Myrmica* colony, thus providing the offspring with their necessary host resources. It was unclear why some *Myrmica* ants keep nesting in the surroundings of *Origanum* plants, thus increasing their chance of being parasitized by *M. arion*. The work by Mannino and colleagues [24] suggested that the ant–plant interaction is maintained by the manipulation achieved through the release of *Origanum* terpenoids which have likely evolved to deter plant enemies directly but could also enhance the plant protection by increasing *Myrmica* ant fidelity and aggressiveness through brain aminergic regulation. 

Interspecific signaling can therefore manipulate the behavior of social insects by influencing their decision-making [94]. However, the diversity of these interactions is so broad that the mechanisms underlying manipulation events are likely to vary significantly within different systems. Still, the aminergic regulation of social insects’ brains remains a promising research scope to fully disentangle the maintenance of these associations. 

## 5. Conclusions

**Figure 6:**
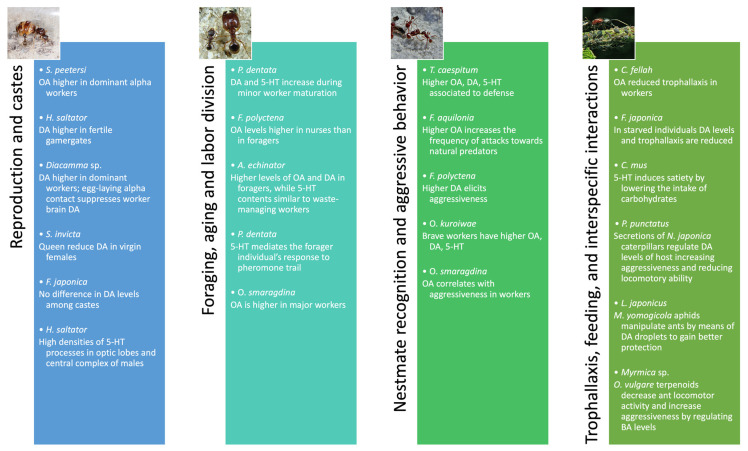
Main functions of BAs in ants. The examples listed in the figure are reported in the following references [24,50,51,52,53,54,57,60,62,66,67,68,75,82,83,84,85,86,87,89,90,91,92].

Although most studies focused on the reproductive role of biogenic amines, primarily assessing the dopaminergic regulation, several other functions have been ascribed to BAs, ranging from division of labor to decision-making (Figure 6). Attempting to generalize: serotonin could regulate metabolic energy and related activities (such as feeding), dopamine modulates reproduction and dominance dynamics, while octopamine is linked to learning and nestmate recognition. All these roles are strictly connected with social behavior and are crucial to maintaining the colony structure.

However, neural circuit anatomy, brain physiology, or gene expression patterns involving BAs are highly conserved throughout insect taxa and are known to regulate similar behavioral responses in both solitary and social organisms [95]. Despite some general similarities, experimental works suggest that a widening array of BA functions have occurred during the transition to eusociality. Irrespective of the level of sociality, dopamine controls the reproductive state, but in eusocial species might have the additional functions to resolve competition between sterile and reproductive castes, for instance, by contributing to maintaining their tasks separated [20]. If octopamine promotes aggression behaviors in solitary species (e.g., [96]), in eusocial insects, it also enhances nestmate recognition (e.g., [54]). The latter ability is pivotal in insect societies to direct aggression only toward intruders while treating colony members benevolently. All three major BAs, serotonin, octopamine, and dopamine, contribute to controlling the metamorphosis of solitary insects (e.g., [97]). Parallelly, in social species, these BA titers vary, mainly increasing [50,66,67], with ages, and can be associated with age-related changes in tasks, from inner to outer duties such as foraging [66]. 

How do insect aminergic circuits and genes for BAs synthesis, regulation, or perception function as pre-adaptations for the evolution of behaviors essential for the switch to social life? Details on proximate and ultimate mechanisms are lacking. However, there is evidence that exogenous administration of serotine promotes the shift from solitary to gregarious behavior in some locusts [98]. In locusts, in particular, aminergic regulation, including dopamine and octopamine, is recognized to control these insects’ sociability levels [99,100,101]. In bees, Hewlett and colleagues [102] provide one of a few instances of a causal role of dopamine in enhancing group cohesion, which along with variation in other BAs, is suggested as a likely driver of sociability in eusocial systems. 

Unfortunately, assessing the direct causal role of any BAs on social or solitary behaviors is a tough challenge. The method used by researchers to manipulate the BA titers in the insect brain might affect the behavior of the target individual by masking the actual effect of the delivered monoamine. BAs could be administered in the diet, but this method helps survey colony-level behaviors and does not allow any control over the dose received by each individual. Ocellar injection, already performed on anesthetized bees [103], implies removing the lens and delivering the BA directly into the brain. Nevertheless, this method is time-consuming, and injection and anesthesia could cause an immune response, ultimately affecting the individual behavior. Topical applications are less invasive methods but are not free from drawbacks. Indeed, the solvent used for administering the BA might induce collateral effects such as those revealed in reducing locomotion in the study by Hewlett and colleagues [102]. In addition, there will be interindividual differences in the amount of administered BA, which can be rapidly transferred from the brain to other body districts. The use of antagonist substances also faces similar issues as those mentioned for the BA administration. 

The methodological shortcomings explain why most of the studies report correlative results. Although these studies contributed to an increase in the knowledge about the effects of BAs on invertebrate behaviors, we believe there should be a call for more research aiming at functionally validating the variation in insect BA in social insects. In detail, we argue that future research should: (i) explore a new non-invasive methodology to artificially modulate the natural BA brain titers avoiding by-product behavioral changes; (ii) clarify the functional role of BA in social and solitary contexts; (iii) dissect the mechanism through which BAs interact or are modulated by a bulk of other molecules and hormones involved in physiological processes; (iv) delve into the relationship between variation in gene expression or epigenetic regulation, BAs, and their action on specific neuronal circuits; (v) expand the array of investigated species, including species showing distinct degrees of sociality and phylogenetic relatedness, to assess patterns of conservation or diversification of BA regulation; (vi) survey multimodal interactions to understand multi-sensory, context-dependent plasticity influencing social behavior. 

To achieve the goals above, advances in single disciplines, such as epigenetics, neuro-anatomy, neurogenetics, and neurochemistry, but primarily their integration with entomology and behavioral ecology, are needed.

Social insects have proved to be perfect models to further delve into the neuroendocrinal regulation of behavior, and BAs seem to be perfect candidates as pivotal factors in promoting the evolution of eusociality in insects.

## Data Availability

Not applicable.
